# Astaxanthin Supplementation Augments the Benefits of CrossFit Workouts on Semaphorin 3C and Other Adipokines in Males with Obesity

**DOI:** 10.3390/nu15224803

**Published:** 2023-11-16

**Authors:** Rashmi Supriya, Sevda Rahbari Shishvan, Movahed Kefayati, Hossein Abednatanzi, Omid Razi, Reza Bagheri, Kurt A. Escobar, Zhaleh Pashaei, Ayoub Saeidi, Shahnaz Shahrbanian, Sovan Bagchi, Pallav Sengupta, Maisa Hamed Al Kiyumi, Katie M. Heinrich, Hassane Zouhal

**Affiliations:** 1 Centre for Health and Exercise Science Research, SPEH, Hong Kong Baptist University, Kowloon Tong, Hong Kong SAR 999077, China; rashmisupriya@hkbu.edu.hk; 2 Department of Physical Education and Sport Science, Science and Research Branch, Islamic Azad University, Tehran 15847-15414, Iran; sevdarahbari1370@gmail.com (S.R.S.); movahed.kefayati@yahoo.com (M.K.); h.abednatanzi@yahoo.com (H.A.); 3 Department of Exercise Physiology, Faculty of Physical Education and Sports Science, Razi University, Kermanshah 94Q5+6G3, Iran; omid.razi.physio@gmail.com; 4 Department of Exercise Physiology, University of Isfahan, Isfahan 81746-73441, Iran; will.fivb@yahoo.com; 5 Department of Kinesiology, California State University, Long Beach, CA 90840, USA; kurt.escobar@csulb.edu; 6 Department of Exercise Physiology, Faculty of Physical Education and Sport Sciences, University of Tabriz, Tabriz 51666-16471, Iran; pashaei.zh@gmail.com; 7 Department of Physical Education and Sport Sciences, Faculty of Humanities and Social Sciences, University of Kurdistan, Sanandaj, Kurdistan 66177-15175, Iran; 8 Department of Sport Science, Faculty of Humanities, Tarbiat Modares University, Tehran 14117-13116, Iran; s.shahrbanian@gmail.com; 9 Department of Biomedical Sciences, College of Medicine, Gulf Medical University, Ajman 4184, United Arab Emirates; dr.sovan@gmu.ac.ae (S.B.); pallav_cu@yahoo.com (P.S.); 10 Department of Family Medicine and Public Health, Sultan Qaboos University, Muscat P.O. Box 35, Oman; maysa8172@gmail.com; 11 Department of Family Medicine and Public Health, Sultan Qaboos University Hospital, Muscat P.O. Box 35, Oman; 12 Department of Kinesiology, Kansas State University, Manhattan, KS 66506, USA; kmhphd@ksu.edu; 13 Research Department, The Phoenix, Manhattan, KS 66502, USA; 14 M2S (Laboratoire Mouvement, Sport, Santé)—EA 1274, Université de Rennes, 35000 Rennes, France; 15 Institut International des Sciences du Sport (2I2S), 35850 Irodouer, France

**Keywords:** nutritional supplements, adipokines, semaphorin 3C, CrossFit workouts, adipose tissue

## Abstract

Regular physical activity and the use of nutritional supplements, including antioxidants, are recognized as efficacious approaches for the prevention and mitigation of obesity-related complications. This study investigated the effects of 12 weeks of CrossFit training combined with astaxanthin (ASX) supplementation on some plasma adipokines in males with obesity. Sixty-eight males with obesity (BMI: 33.6 ± 1.4 kg·m^−2^) were randomly assigned into four groups: the control group (CG; *n* = 11), ASX supplementation group (SG; *n* = 11), CrossFit group (TG; *n* = 11), and training plus supplement group (TSG; *n* = 11). Participants underwent 12 weeks of supplementation with ASX or placebo (20 mg/day capsule daily), CrossFit training, or a combination of both interventions. Plasma levels of semaphorin 3C (SEMA3C), apelin, chemerin, omentin1, visfatin, resistin, adiponectin, leptin, vaspin, and RBP4 were measured 72 h before the first training session and after the last training session. The plasma levels of all measured adipokines were significantly altered in SG, TG, and TSG groups (*p* < 0.05). The reduction of resistin was significantly higher in TSG than in SG (*p* < 0.05). The plasma levels of omentin1 were significantly higher in both training groups of TG and TSG than SG (*p* < 0.05), although such a meaningful difference was not observed between both training groups (*p* > 0.05). Significant differences were found in the reductions of plasma levels of vaspin, visfatin, apelin, RBP4, chemerin, and SEMA3C between the SG and TSG groups (*p* < 0.05). The study found that a 12-week intervention using ASX supplementation and CrossFit exercises resulted in significant improvements in several adipokines among male individuals with obesity. Notably, the combined approach of supplementation and training had the most pronounced results. The findings presented in this study indicate that the supplementation of ASX and participation in CrossFit exercise have the potential to be effective therapies in mitigating complications associated with obesity and enhancing metabolic health.

## 1. Introduction

Obesity is characterized by an excessive accumulation of adipose tissue and is strongly linked to the development and progression of several metabolic disorders [1,2]. Accumulated adipose tissue not only acts as a reservoir for excess energy, but also functions as an endocrine organ that releases molecular proteins known as adipokines [1,3]. Of these adipokines, leptin, resistin, visfatin, apelin, retinol binding protein4 (RBP4), vaspin, and chemerin are associated with obesity, while others such as adiponectin and omentin1 have a negative correlation. These adipokines are involved in various physiological processes such as metabolism and glucose homeostasis, oxidative stress, and the pathophysiology of obesity [4,5,6]. Leptin exerts its effects on hunger reduction and the restoration of energy balance by acting on central processes, namely by blocking certain leptin-sensitive neurons such as neuropeptide Y and proopiomelanocortin neurons, hence promoting energy homeostasis [7,8].

Engaging in regular physical activity is a potent strategy for enhancing general well-being, preventing and decreasing obesity, and alleviating the adverse health consequences linked to excessive adipose tissue [5,9]. CrossFit is an exercise regimen characterized by the use of diverse functional movements derived from several athletic disciplines, including weightlifting, gymnastics, and powerlifting. These movements are performed in rigorous sessions that emphasize high-intensity training [10]. Previous studies have confirmed the positive effects of CrossFit training on physiological and fitness factors (e.g., body composition, cardiovascular/respiratory fitness, strength, flexibility, power, and balance) [11,12].

At the present time, data are insufficient on the impact of CrossFit training on the adipokines that are the subject of investigation in the current study. However, literature exists on other modes of training for various adipokines [5,13,14,15,16,17]. For example, jogging and step aerobic exercise increased leptin and interleukin-15 (IL-15) while decreasing resistin in overweight women [18]. Jung et al. [15] indicated a significant decrease in blood leptin levels after 12 weeks of engaging in moderate-intensity exercise—namely brisk walking—among both obese men and females. Ouerghi et al. [19] showed that plasma levels of omentin-1 increased after 8 weeks of high-intensity interval training (HIIT) in obese participants, along with reduced obesity, blood lipids, and insulin sensitivity. However, others have found no significant changes in omentin-1 after a training period [20,21]. 

Antioxidant supplementation can be used to attenuate the negative effects of oxidative stress [22]. Astaxanthin (3,3′-dihydroxy-B, B-carotene4, 4′-dione), which is derived from *Haematoccus pluvialis* algae, has been shown to reduce the effects of oxidative stress on lipid metabolism [23]. Systematic review and meta-analysis studies revealed that Astaxanthin (ASX) supplementation was associated with a decrease in insulin resistance and oxidative stress, an increase in antioxidant capacity and mitochondrial biogenesis in obesity, as well as improvements regarding diabetes, cardiovascular disease, neurodegenerative disorders, chronic inflammatory disease, and some cancers [24,25]. Furthermore, it also improves lipid metabolism [23,26,27]. Moreover, ASX supplementation improves insulin resistance in obese mice by modulating insulin signaling and activating mitochondrial energy metabolism via pathways for AMP-activated protein kinase (AMPK) and peroxisome proliferator-activated receptor γ coactivator1a (Pgc1a) in skeletal muscles [28]. Although there is less available evidence about the precise mechanism by which ASX supplementation acts, it is postulated that the favorable effects of ASX may be attributed to its impact on the secretion of adipokines, akin to other bioactive chemicals such as capsaicin. Although a recent review study found that combined ASX supplementation and exercise did not improve exercise performance [29], it is unknown whether a combination of ASX supplementation and exercise produces beneficial effects on metabolic health in obese individuals. Furthermore, the effects of ASX supplementation on adipokines in the present study are currently unclear [29]. We have previously found that ASX supplementation and CrossFit training improved body composition, metabolic profiles, anthropometric measurements, cardio-respiratory function, and some adipokines (i.e., Cq1/TNF-related protein 9 and 2 [CTRP9 and 2] and growth differentiation factor 8 and 15 [GDF8 and 15]) [30], but the effect of CrossFit training on other adipokines (semaphorin 3C (SEMA3C), apelin, chemerin, omentin1, visfatin, resistin, adiponectin, leptin, vaspin, and RBP4) is unexplored. On the other hand, the current study hypothesized that CrossFit training and ASX supplementation has a positive effect on SEMA3C, apelin, chemerin, omentin1, visfatin, resistin, adiponectin, leptin, vaspin, and RBP4 in males with obesity. Therefore, this study aimed to investigate the effect of 12 weeks of CrossFit training combined with ASX supplementation on SEMA3C, apelin, chemerin, omentin1, visfatin, resistin, adiponectin, leptin, vaspin, and RBP4 in males with obesity.

## 2. Methods

Participant recruitment has been described previously (see [30]). Study inclusion criteria were body mass index (BMI) > 30 kg·m^−2^, lack of regular physical exercise in the last six months, absence of cardiovascular, metabolic, or endocrine disorders, and no alcohol intake. The research excluded individuals who had joint disorders, physical limitations, or were using prescription drugs and supplements that might potentially impact muscle and adipose tissue metabolism [31]. The participants were originally presented with a thorough explanation of the research protocols. Subsequently, all participants had a medical examination conducted by a physician and clinical exercise physiologist on their first visit. Additionally, they were required to sign a written consent form and the Physical Activity Readiness Questionnaire (PARQ) [32]. The study was approved by the National Research and Ethics Committee (Ethics code: IR.IAU.DAMGHAN.REC.1401.035) and the Iranian Registry of Clinical Trials (IRCTID: IRCT20151228025732N76). The procedures were conducted in accordance with the most recent version of the Declaration of Helsinki [33].

### 2.1. Experimental Design

**Figure 1:**
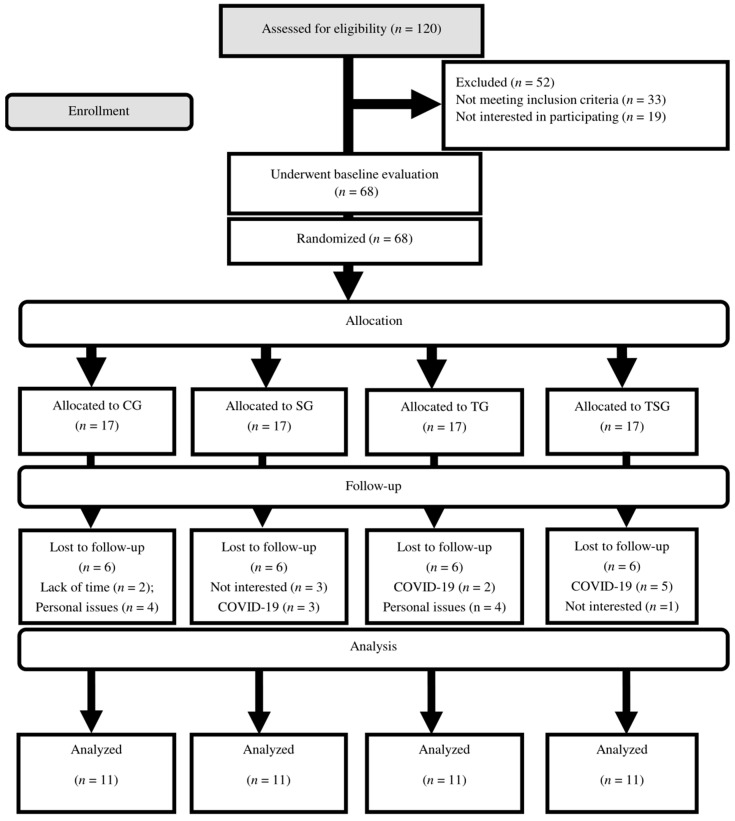
Flow chart of the participant recruitment.

Participants were familiarized with the entire study procedure one week prior to the initiation of the main training protocol. Basic measures including height and body weight were assessed (see [30]). Then, 68 eligible participants (age: 27 ± 8 yrs.; height: 167.8 ± 3.1 cm; body weight: 94.7 ± 2.0 kg, BMI: 33.6 ± 1.4 kg·m^−2^) were randomly divided into four groups: control group (CG; *n* = 17), ASX supplement group (SG; *n* = 17), CrossFit group (TG; *n* = 17), and training plus supplement group (TSG; *n* = 17). The flow of participant recruitment is outlined in Figure 1. During the study, six individuals per group declined to participate in the remaining protocol procedures due to medical, job, or lack of interest reasons. Each group (collectively, *n* = 11) received instructions on performing the training protocols during the third session. Following baseline measurements, the two training groups (TG and TSG) attended CrossFit training (3 sessions/week) for 12 weeks. The control group participants were provided with instructions to maintain their existing lifestyles throughout the duration of the trial. The measurements for the study were conducted simultaneously, with a time difference of around one hour, under similar climatic circumstances, with a temperature of around 20 °C, and a humidity level of approximately 55%. The pre-and post-test measures were conducted 48 h before initiation and after the end of the last training session, respectively.

### 2.2. Training Protocols

In this study, the HIFT program was used, which included CrossFit training in 36 sessions, each session lasting 60 min and performed three times a week. All HIFT sessions were led by a CrossFit Level 1-certified trainer. The first two sessions were designed as an introduction to common movements used in HIFT (air squat, overhead squat, front squat, press, push press, push jerk, deadlift, sumo deadlift high pull, and the medicine ball clean). Starting on the third day, each HIFT session consisted of 10–15 min of stretches and warm-ups; 10–20 min of instruction and practicing methods and movements; and 5–30 min of the workout of the day (WOD), conducted at a vigorous intensity according to each individual’s fitness level. Workout modality components included aerobics (e.g., running, jumping rope), body weight (e.g., pull-ups, squats), and weightlifting (e.g., front squats, kettlebell swings) exercises that were continuously varied using the CrossFit training template [34] in single, couplet, or triplet. All weights and movements were prescribed and recorded separately for every HIFT participant [35]. Depending on the structure of the WOD, timings to complete the WOD, rounds, and repetitions performed on the WOD, the weights used and any necessary modifications to the scheduled workout were also noted for each participant. For the HIFT group as a whole, average times for each WOD and the total average WOD time per week were calculated.

### 2.3. Astaxanthin Supplementation Protocol

The participants in the SG and TSG were randomly allocated to receive a daily dose of 20 mg of ASX capsule (manufactured by Marine Product Tech. Inc., Seongnam, Republic of Korea) or a placebo consisting of a 20 mg dose of a raw corn starch capsule. This administration took place once daily, with breakfast, for a duration of 12 weeks [36].

### 2.4. Nutrient Intake and Dietary Analysis

To evaluate changes in dietary habits, a set of three-day food records (consisting of two weekdays and one weekend day) was obtained before and after the research. Every meal item was individually inputted into Diet Analysis Plus version 10 (Cengage, Boston, MA, USA) in order to determine the total calorie consumption and the relative distribution of energy derived from fats, proteins, and carbohydrates [31].

### 2.5. Blood Markers

The procedure of blood testing was performed under standard conditions between 8 and 10 a.m. Samples for fasting blood sugar were drawn from the right arm 12 and 72 h prior to the first exercise session and again at 72 h after the last session. EDTA-containing tubes were used to transfer the blood samples, which were centrifuged for 10 min at 3000 rpm and stored at −70 °C. Plasma resistin was measured with an ELISA kit (Biovendor, Czech Republic, Catalogue No: RD191016100. Sensitivity: 0.012 ng/mL. Intra-CV = 5.9%, inter-CV = 7.6%). Plasma leptin was measured with an ELISA kit (Biovendor, Czech Republic, Catalogue No: RD191001100. Sensitivity: 0.2 ng/mL. Intra-CV = 5.9%, inter-CV = 5.6%). Plasma adiponectin was measured with an ELISA kit (Biovendor, Czech Republic, Catalogue No: RD195023100. Sensitivity: 26 ng/mL. Intra-CV = 4.9%, inter-CV = 6.7%). Plasma visfatin was measured with an ELISA kit (Cusabio, China, Catalog No: CSB-E08940h. Sensitivity: 0.156 ng/mL. Intra-CV = < 8%, inter-CV = < 10%). Plasma vaspin was measured with an ELISA kit (Biovendor, Czech Republic, Catalogue No: RD191097200R. Sensitivity: 0.01 ng/mL. Intra-CV = 7.6%, inter-CV = 7.7%). Plasma RBP-4 was measured with an ELISA kit (R&D Systems, USA, and Catalogue No: DRB400. Sensitivity: 0.628 ng/mL. Intra-CV = 7%, inter-CV = 8.6%). Plasma apelin was measured with an ELISA kit (Phoenix Pharmaceuticals, USA, and Catalogue No: EK-057-23. Sensitivity: 0.07 ng/mL. Intra-CV = < 10%, inter-CV = < 15%). Plasma omentin-1 was measured with an ELISA kit (Biovendor, Czech Republic, Catalogue No: RD191100200R. Sensitivity: 0.5 ng/mL. Intra-CV = 3.7%, inter-CV = 4.6%). Plasma chemerin levels were determined using a commercially available ELISA kit (Biovendor, Czech, The intra-assay coefficient of variation of chemerin was 5.1%). The plasma levels of SEMA3C (MBS037239, MBS2883689, MyBioSource, San Diego, CA, USA) were measured by commercially available enzyme-linked immunosorbent assay (ELISA) kits.

### 2.6. Statistical Analysis

G-power 3.1.9.2 software was used to calculate the sample size, and based on the previous study, it was determined that there was a significant effect of combined training on reducing leptin levels in overweight and obese males [37]. This study utilized the equation for effect size (ES) to determine the impact of combined aerobic and resistance training. In the present study, based on α = 0.05, a power (1-β) of 0.95, and an effect size (ES) = 1 ((5.4 − 3.6)/1.65), a total sample size of at least 20 participants (*n* = 5 per group) was required to detect significant changes in leptin levels. Nevertheless, given the absence of prior studies investigating the impact of CrossFit on the measured adipokines in the current investigation, along with the potential hindrance of COVID-19 on training and adherence to supplementation, it was deemed necessary to increase the sample size (*n* = 17) to maintain the statistical power of the study. Descriptive statistics (means ± standard deviation) were used to describe all the data. The Shapiro Wilk test and two-way ANOVA were used to assess the normality of the data and determine the group x time interaction, respectively. One-way ANOVA and Fisher LSD post-hoc tests were used for the evaluation of the baseline data of the four groups. In addition, pairwise comparisons were used to determine mean differences when a significant difference between groups was detected by ANOVA. Additionally, effect sizes (ES) were reported as partial eta-squared. In accordance with Hopkins et al. (2009), ES was considered trivial (<0.2), small (0.2–0.6), moderate (0.6–1.2), large (1.2–2.0), and very large (2.0–4.0). Statistical significance was determined using a *p*-value threshold of less than 0.05. Pearson’s linear regressions were performed with a 95% confidence interval (CI). Values ranging from 0 to 0.3 (or 0 to −0.3) are indicative of a weak positive (negative) linear relationship through a shaky linear rule. Values ranging from 0.3 to 0.7 (−0.3 to −0.7) are indicative of a moderate positive (negative) correlation. Values falling within the range of 0.7 to 1.0 (−0.7 and −1.0) are indicative of a strong positive (negative) correlation [38]. The statistical analyses were conducted using SPSS 26, while the generation of figures was carried out using GraphPad Prism (version 8.4.3).

## 3. Results

### 3.1. Compliance, Adverse Events, and Nutrient Intakes

Participant compliance was considered when ≥80% of the supplements were consumed. Six participants from each group withdrew due to personal reasons and COVID-19. No adverse events were reported from both training and supplementation procedures. Also, no changes in nutrient intakes were observed throughout the study (Table 1).

### 3.2. Adipokines

**Figure 2:**
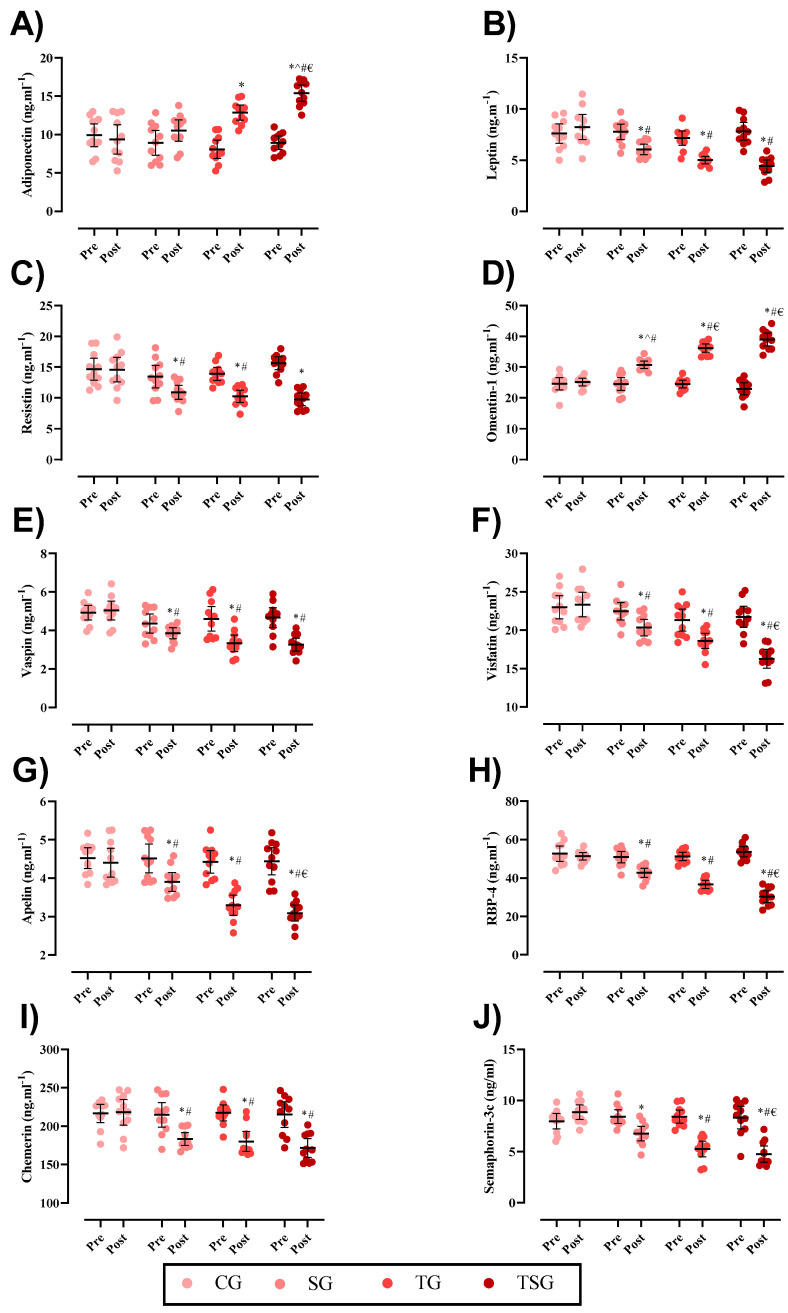
Changes in adipokines throughout the intervention. (**A**) Adiponectin; (**B**) Leptin; (**C**) Resistin; (**D**) Omentin; (**E**) Vaspin; (**F**) Visfatin; (**G**) Apelin; (**H**) Retinol binding protein 4 (RBP-4); (**I**) Chemerin; (**J**) Semaphorin-3c. *n* = 11 per group, error bars represent a 95% confidence interval (CI). * Significantly different from pre-test; # Significantly different than CG; ^ Significantly different than TG; € Significantly different than SG.

Changes in adipokines throughout the intervention are shown in Figure 2. Baseline levels of adiponectin (*p* = 0.20), leptin A (*p* = 0.58), resistin (*p* = 0.12), omentin1, (*p* = 0.46), vaspin (*p* = 0.40), visfatin (*p* = 0.24), apelin (*p* = 0.94), RBP4 (*p* = 0.45), chemerin (*p* = 0.89), and SEMA3C (*p* = 0.81) were not significantly different between groups. Following the 12-week intervention, there were significant group × time interactions for adiponectin (*p* = 0.0001, η^2^ = 0.48, statistical power = 0.999), leptin (*p* = 0.0001, η^2^ = 0.49, statistical power = 0.998), resistin (*p* = 0.0001, η^2^ = 0.40, statistical power = 0.993), omentin-1 (*p* = 0.0001, η^2^ = 0.74, statistical power = 1.00), vaspin (*p* = 0.0001, η^2^ = 0.30, statistical power = 0.936), visfatin (*p* = 0.0001, η^2^ = 0.35, statistical power = 0.937), apelin (*p* = 0.0001, η^2^ = 0.43, statistical power = 0.997), RBP4 (*p* = 0.0001, η^2^ = 0.70, statistical power = 1.00), chemerin (*p* = 0.0001, η^2^ = 0.29, statistical power = 0.856), and SEMA3C (*p* = 0.0001, η^2^ = 0.51, statistical power = 1.00).

In comparison to the baseline, post-intervention values for adiponectin (*p* = 0.55), leptin (*p* = 0.22), resistin (*p* = 0.93), omentin-1 (*p* = 0.58), vaspin (*p* = 0.70), visfatin (*p* = 0.69), Apelin (*p* = 0.48), RBP4 (*p* = 0.42), chemerin (*p* = 0.76) and SEMA3C (*p* = 0.10) were not significantly different in the CG. Changes in adiponectin (*p* = 0.80) and vaspin (*p* = 0.09) were not significantly different in the SG. Post-test values were significantly different in comparison to the baseline for the rest of the variables in the SG, and also in the TG and TSG for all of the adipokines (*p* = 0.001) in this study.

The increases in plasma adiponectin levels after 12 weeks of intervention in the TG (*p* = 0.0001) and TSG (*p* = 0.0001) were significant, but not in the SG (*p* = 0.10) in comparison to the CG. The differences were non-significant between the TSG and TG (*p* = 0.19), while the differences between the SG and TG (*p* = 0.01) and the SG and TSG (*p* = 0.0001) were statistically significant (Figure 2A). The changes in plasma leptin levels after 12 weeks of intervention in the SG (*p* = 0.002), TG (*p* = 0.0001), and TSG (*p* = 0.0001) were significantly lower in comparison to the CG; the differences between the SG and TSG (*p* = 0.02) were statistically significant (Figure 2B).

Plasma levels of resistin were significantly decreased post-test compared to the baseline in the SG (*p* = 0.033), TG (*p* = 0.003), and TSG (*p* = 0.0001). The differences between the SG and TSG (*p* = 0.005), TSG and CG (*p* = 0.0001), and TG and CG (*p* = 0.0001) were statistically significant (Figure 2C). The plasma levels of omentin-1 were significantly increased among three interventional groups of the SG (*p* = 0.001), TG, and TSG (*p* = 0.0001) in comparison with CG. There were also significant differences in the TG and TSG (*p* = 0.001) compared with SG, as well as between the TG and TSG (*p* = 0.007) (Figure 2D). The plasma levels of vaspin were significantly reduced only in 12-week training groups of the TG (*p* = 0.002) and TSG (*p* = 0.001), while supplementation with (SG) was not different (*p* = 0.14). Vaspin levels were different between the SG and TSG (*p* = 0.034), but not between the TG and TSG (*p* = 0.74), nor between the SG and TG (*p* = 0.06) (Figure 2E). Plasma levels of visfatin were changed in the TG (*p* = 0.018) and TSG (*p* = 0.0001), while there was no difference in the SG (*p* = 0.054). There were no differences between the SG and CG (*p* = 0.64), while the differences between the SG and TSG (*p* = 0.011) and SG and TG (*p* = 0.035) were statistically different (Figure 2F). Plasma levels of apelin were meaningfully reduced in the TG and TSG (*p* = 0.0001), but not in the SG (*p* = 0.051). There were no differences in apelin between the TG and TSG (*p* = 0.37), but there were differences between the SG and TSG (*p* = 0.004) and also between the SG and TG (*p* = 0.038) (Figure 2G). The alterations of plasma RBP4 level were significant in three interventional groups; the SG (*p* = 0.007), TG, and TSG (*p* = 0.0001) following the 12-week interventions. There were, otherwise, significant differences between the SG and TG (*p* = 0.009), SG and TSG (*p* = 0.0001), as well as between the TG and TSG (*p* = 0.001) (Figure 2H). Similarly, 12 weeks of training and/or ASX supplementation altered chemerin in the SG (*p* = 0.017), TG, and TSG (*p* = 0.001). However, there were not any significant differences between the SG and TG (*p* = 0.30), SG and TSG (*p* = 0.31), nor between the TG and TSG (*p* = 0.97) in the changes of chemerin (Figure 2I). The plasma levels of SEMA3C were significantly reduced in three groups of the SG (*p* = 0.002), TG, and TSG (*p* = 0.0001) following 12-week ASX supplementation and CrossFit training. Based on the results of the post-hoc test, there were non-significant differences between the SG and TG (*p* = 0.057) and the TG and TSG (*p* = 0.58), but the changes between SG and TSG (*p* = 0.016) were statistically significant (Figure 2J).

### 3.3. Weight and BMI

There were no between-group differences in baseline values for weight (*p* = 0.46) and BMI (*p* = 0.57). There were significant group X time interactions for weight (*p* = 0.0001, η^2^ = 0.46, statistical power = 0.999) and BMI (*p* = 0.002, η^2^ = 0.30, statistical power = 0.998) (Table 1).

Body weight reductions after 12 weeks were significant in the SG (*p* = 0.008), TG (*p* = 0.0001), and TSG (*p* = 0.0001) but not in the CG (*p* = 0.32). Furthermore, the post-hoc test for bodyweight shows that after 12 weeks there were significant changes in the CG compared to the TG (*p* = 0.004) and TSG (*p* = 0.0001), and in the TSG compared to the TG (*p* = 0.01) and SG (*p* = 0.0001), while other changes were not significant (*p* > 0.05) (Table 1).

Changes in BMI after 12 weeks were significantly decreased in the SG (*p* = 0.019), TG (*p* = 0.0001), and TSG (*p* = 0.0001) but not in the CG (*p* = 0.37). BMI changes after 12 weeks were significantly decreased in the TG (*p* = 0.016) and the TSG (*p* = 0.0001) compared to the CG. The differences induced by training were significant between the TG and TSG (*p* = 0.007) and between the SG and TSG (*p* = 0.007), while all other differences in BMI between the groups were not significant (*p* > 0.05) (Table 1).

**Figure 3:**
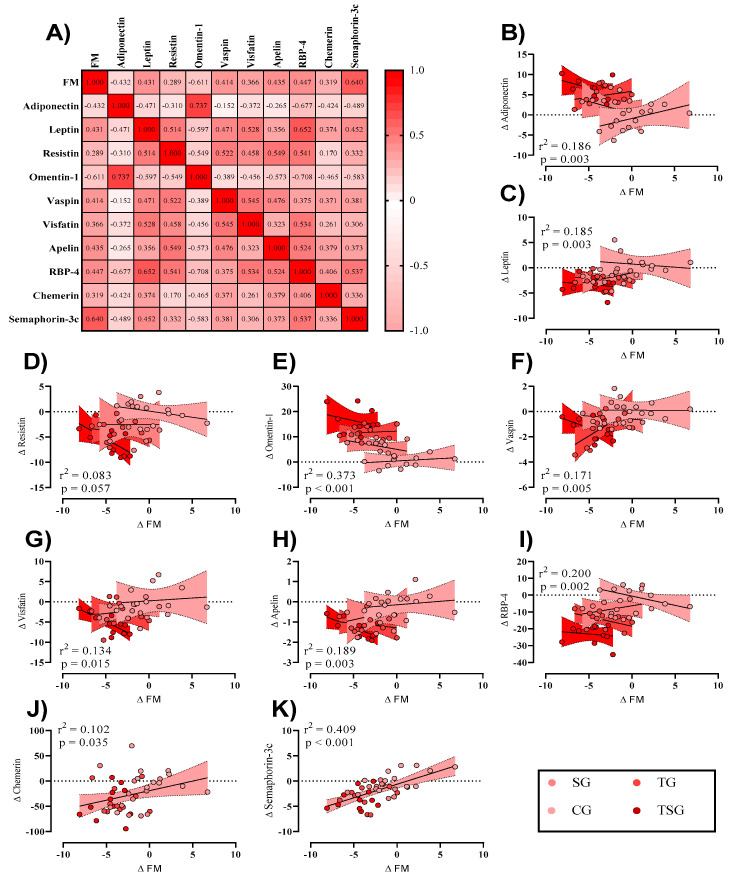
(**A**) Correlation matrix of Δ FM and adipokines, r values as shown. The key indicates the magnitude of r (red = −1 or 1, white = 0). (**B**–**K**) linear regression (Pearson’s) of Δ (adipokine) as a function of Δ FM (kg). Linear regression is indicated by the solid black line, and 95% confidence intervals are indicated by red zones.

To investigate any potential relationships between training-induced changes in fat mass (Δ FM) and changes in adipokines (Δ marker, independently of groups), initially, a correlation matrix was generated (Figure 3A). Adiponectin (Figure 3B) and omentin1 (Figure 3E) showed moderate negative relationships with Δ FM, while leptin (Figure 3C), vaspin (Figure 3F), visfatin (Figure 3G), apelin (Figure 3H), RBP4 (Figure 3I), chemerin (Figure 3J), and SEMA3C (Figure 3K) showed a moderate positive relationship. Also, resistin (Figure 3D) showed a weak positive relationship. For linear regression of individual Δ (adipokine) as a function of Δ FM, data were examined by the extra sum-of-squares F test to first consider if pooled data could be considered as a single model. Only chemerin and SEMA3C were considered a single group. All data except for resistin showed a significant relationship with changes in FM (a trend was observed; *p* = 0.057).

## 4. Discussion

Adipokines play a key role in cardiometabolic health status, and circulating levels are altered in obese states [39]. This study demonstrated that 12 weeks of CrossFit training and ASX supplementation, separately and in combination, can improve circulating adipokines levels in obese men. The combination of CrossFit and ASX supplementation overall led to greater changes in measured outcomes compared to each intervention alone. In our previous study, it was shown that CrossFit training and ASX supplementation decreased the plasma levels of GDF8, GDF15, CTRP2, and CTRP9. We also showed that CrossFit training and ASX supplementation increases high-density lipoprotein (HDL) and VO_2peak_, and decreases low-density lipoprotein (LDL), total cholesterol (TC), TG, and insulin resistance [30]. This is the first investigation using CrossFit training as a mode of exercise as well as in combination with ASX supplementation on SEMA3C, apelin, chemerin, omentin1, visfatin, resistin, adiponectin, leptin, vaspin, and RBP4 in males with obesity.

Adiponectin has been shown to be inversely associated with insulin resistance and obesity [40]. In the present study, it was shown that CrossFit and ASX supplementation alone caused an increase in adiponectin in obese people, while this increase was greater in the group that took CrossFit training and ASX supplementation together. Some studies confirm the results of our research [41,42,43], while others do not show any change in adiponectin levels following acute exercise [44,45]. This disagreement may have been related to the laboratory protocols used [40]. Although the mechanism of action of CrossFit training in increasing plasma adiponectin levels is not well understood, the secretion of catecholamines, B-adrenergic receptors activity, and reduction of tumor necrosis factor-alpha (TNFa) may play a role [40]. Also, the increase in HDL and decrease in LDL, cholesterol, TG, and insulin resistance in our research [30] can be one of the reasons for the increase in adiponectin. Previous literature has also shown ASX supplementation increased serum adiponectin levels (~26%) in adults with mild hyperlipidemia [46]. The mechanisms underlying the effect of ASX supplementation on adiponectin are unclear, but one of the possible reasons could be reductions in TNFa through the activity of the peroxisome proliferator-activated receptor gamma (PPARγ) pathway [46].

Plasma leptin levels also decreased in each intervention group after 12 weeks. One piece of research agrees with the results of this study [41,44,47], while others have shown no effect of exercise in altering leptin levels [48,49,50]. These conflicting results may be due to differences in exercise protocols. The decrease in leptin levels following CrossFit training and ASX supplementation is likely related to the reduction of fat mass [40], which we previously published for this sample [30]. With the advancement of technology along with the improvements in living conditions, chronic diseases such as diabetes have increased as a result of low physical activity levels and improper nutrition. As a result of these processes, it is necessary to find safe interventions without complications [51]. Feng et al. [52] observed that ASX supplementation led to an improvement of insulin sensitivity and glucose tolerance through the suppression of inflammation, which reduced the symptoms of diabetes. Due to the strong antioxidant role of ASX supplementation, its anti-obesity and anti-inflammatory roles have been shown. For example, mechanisms include improving glucose metabolism, lowering blood pressure, improving redox imbalance in lymphocytes, and protecting β cells in the pancreas due to ASX supplementation has proven anti-diabetic and anti-obesity properties [51]. ASX supplementation also accelerates the metabolism of TG and HDL, reduces the incidence of cardiovascular disease, and increases the level of adiponectin, which plays an important role in regulating blood glucose [53]. The researchers observed that ASX reduced the production of nitric oxide (NO), leading to a reduction in insulin resistance through increased serine phosphorylation of insulin receptor substrate 1 (IRS1). In their study, Xia et al. [24] showed that ASX supplementation leads to a decrease in the size of fat cells through the activation of PPARγ. This leads to a decrease in plasma free fatty acid (FFA) levels, which confirms the results of Hussein et al. [54]. Aoi et al. [23] observed in their study that ASX supplementation leads to a reduction in the oxidative damage of carnitine palmitoyl transferase I (CPTI). This factor plays an important role in the oxidation of fatty acids in the mitochondrial membrane of muscle tissue. Contradictory results have been observed regarding the effect of ASX supplementation; some showed a non-significant increase, and others showed no change or decrease [23]. Hossein et al. [54] showed that long-term ASX supplementation (50 mg/kg/day) led to an increase in plasma adiponectin levels. Yoshida et al. [46] also observed these changes. Further research should be performed to determine the most effective dose and duration of treatment to increase blood adiponectin levels under different conditions.

In addition to reducing oxidative stress, ASX supplementation led to an increase in the serum levels of adiponectin in obese rats [55]. The effective mechanism of this process leads to the suppression of liver cancer in obese people [46]. Due to increased hormone-sensitive lipase activity in response to CrossFit training in obese participants, adiponectin levels increased significantly, and this led to body fat regulation. The important role of leptin is in energy balance and appetite control, and as a result, the level of this hormone is low in obese people. The cause of increased leptin in obese people can be attributed to resistance to leptin [56]. The normal level of this hormone is between 1 and 15 ng/mL in normal people, and more than 30 ng/mL in obese people [57]. Chronic high-intensity training has led to a decrease in blood leptin levels in obese participants [58]. The mechanism of this reduction was the reduction of body fat levels. Exercise leads to a decrease in leptin levels and an increase in adiponectin levels [56]. Some of the different results obtained can be due to different training protocols, variables under investigation, and more. However, in general, it has been observed that long-term training has a greater effect on leptin and adiponectin levels [59,60]. Due to its intensity and sufficient duration, CrossFit training leads to a decrease in body weight and a change in body composition through a negative balance created between energy intake and energy consumption [61]. Adiponectin and leptin are among the most well-known cytokines, which are secreted by adipose tissue and play an important role in metabolic and anti-inflammatory processes. In many chronic diseases, low levels of adiponectin and high levels of leptin play an important role in disease progression [40,62].

In the present study, after CrossFit workouts, the level of plasma omentin-1 significantly increased [63]. The effect of aerobic or resistance training on omentin-1 is conflicting, with studies showing increased levels [64,65] or unchanged levels [60]. In our previously published study [30] and other studies [59,60], the mechanism of increase in the level of omentin-1 has been shown, due to the reduction of body weight and improvement of cardiometabolic status. The level of omentin1 decreases in proportion to the increase in obesity [66], and the use of interventions to reduce body weight such as diet and active lifestyle leads to an increase in the level of omentin-1 [67]. This leads to weight loss, and as a result, the level of omentin1 increases [21,68]. Omentin is produced in adipose tissue; it seems that myokines released by muscle cells in response to positive exercise affect omentin1 levels [68].

The possible mechanism of visfatin reduction in different studies may be due to the intensity of training and the amount of changes in body weight and body fat volume. Also, vaspin is an adipokine that improves insulin sensitivity as a result of reducing body fat due to exercise, leading to an increase in its serum levels [69]. SEMA3C is a protein that plays an important role in the development of nervous, cardio-respiratory, kidney systems, and various oncogenesis [70]. This adipokine is secreted from subcutaneous fat tissues, and its level of secretion is related to obesity level, fat cell morphology, and weight changes [70]. Few studies have been performed on the effect of exercise training on the SEMA3C level. Limited research has found a decrease in serum SEMA3C levels following long-term training, and this decrease was significantly associated with improvements in body weight and body fat levels [70]. Also, the increase in HDL and decrease in LDL, TG, and insulin resistance in our research [30] can be one of the reasons for the decreased SEMA3C level. The current investigation shows that a 12-week regimen of CrossFit exercise training, in conjunction with a 20 mg dosage of ASX supplementation, resulted in a substantial decrease in adipokines that are directly associated with obesity. The observed enhancement was more pronounced in the group that had concurrent Crossfit training and ASX supplementation.

### Study Limitations

There are various limitations inherent in our investigation. Initially, the processes behind the potential enhancement of adipokine levels by bioactive constituents of ASX were not determined. Furthermore, the generalizability of our research is limited due to the exclusion of females in the enrollment of patients. Another limitation of our study is the lack of measurement of adipokine levels. Furthermore, it should be noted that the current body of research on the impact of ASX and CrossFit training on adipokines is minimal. Consequently, the precise processes behind this relationship remain undetermined. Therefore, further investigation is needed to elucidate potential pathways.

## 5. Conclusions

Our research presents novel insights regarding the impact of a combined regimen of CrossFit training and ASX supplementation on adipokines in males with obesity. Our data suggest that non-drug strategies such as ASX supplementation with CrossFit training can reduce SEMA3C, apelin, chemerin, visfatin, RBP4, resistin, vaspin, and leptin, and increase adiponectin and omentin1 in males with obesity. Consequently, individuals with obesity are recommended to include CrossFit exercise in their physical activity regimen and use ASX supplements in their dietary intake.

## Figures and Tables

**Table 1:** Mean (±SD) values of BMI, body weight, and nutritional intake throughout the intervention.

	CG		SG		TG		TSG	
	Pre	Post	Pre	Post	Pre	Post	Pre	Post
Energy (kcal/day)	2260 ± 47	2269 ± 56	2278 ± 101	2149 ± 100	2269 ± 117	2141 ± 117	2273 ± 157	2129 ± 126
CHO (g/day)	281 ± 31.4	283 ± 33.3	279.4 ± 27.1	261 ± 27.5	289 ± 48.6	261 ± 39.2	288 ± 38.6	259 ± 29.1
Fat (g/day)	82.2 ± 11.0	81 ± 9.8	86.5 ± 10.7	75 ± 11.2	83.4 ± 12.4	73.1 ± 11.2	80.8 ± 13.87	70.2 ± 11.3
Protein (g/day)	104 ± 12.0	106 ± 11.3	101 ± 13.5	93 ± 12.6	103 ± 14.8	94 ± 11.7	102 ± 14.5	90 ± 13.5
Body Weight (kg)	95.3 ± 1.8	92.1 ± 2.1	94.2 ± 2.6	90.1 ± 2.3 ^a^	94.3 ± 0.9	90.1 ± 2.3 ^a,b^	95.1 ± 1.9	88.2 ± 2.3 ^a,b,ab^
BMI (kg/m^2^)	34.1 ± 2.5	33.7 ± 1.4	33.2 ± 1.4	32.4 ± 1.6 ^a,b^	33.5 ± 1.7	32.1 ± 1.5 ^a,b^	33.8 ± 1.2	31.8 ± 0.6 ^a,b,ab^

CG: Control group; SG: Supplement group; TG: Training group; TSG: Training + Supplement group BMI: Body Mass Index. ^a^ Indicates significant differences compared to the pre-values (*p* < 0.05). ^b^ Significant differences compared to the control group (*p* < 0.05). ^ab^ Significant interaction between time and groups (*p* < 0.05).

## Data Availability

The datasets generated and/or analyzed during the current study are not publicly available but are available from the corresponding author upon reasonable request.
